# Novel High-Throughput Fluorescence-Based Assay for the Identification of Nematocidal Compounds That Target the Blood-Feeding Pathway

**DOI:** 10.3390/ph15060669

**Published:** 2022-05-27

**Authors:** Anthony Marchand, Joyce W. M. Van Bree, Aya C. Taki, Mati Moyat, Gerardo Turcatti, Marc Chambon, Adam Alexander Thil Smith, Rory Doolan, Robin B. Gasser, Nicola Laraine Harris, Tiffany Bouchery

**Affiliations:** 1 Laboratory of Intestinal Immunology, École Polytechnique Fédérale de Lausanne (EPFL), 1015 Lausanne, Switzerland; anthony.marchand@epfl.ch (A.M.); mati.moyat@gmail.com (M.M.); nicola.harris@monash.edu (N.L.H.); 2 Department of Immunology and Pathology, Alfred Medical Research and Education Precinct (AMREP), Monash University, Melbourne, VIC 3004, Australia; joyce.vanbree@wur.nl; 3 Melbourne Veterinary School, The University of Melbourne, Melbourne, VIC 3052, Australia; aya.taki@unimelb.edu.au (A.C.T.); robinbg@unimelb.edu.au (R.B.G.); 4 Biomolecular Screening Facility, École Polytechnique Fédérale de Lausanne (EPFL), 1015 Lausanne, Switzerland; gerardo.turcatti@epfl.ch (G.T.); marc.chambon@epfl.ch (M.C.); 5 Metabolomics Laboratory, Baker Heart and Diabetes Institute, Melbourne, VIC 3004, Australia; alex.smith@fmi.ch; 6 Hookworm Immuno-Biology Laboratory, Swiss Tropical and Public Health Institute, 4123 Allschwill, Switzerland; rory.doolan@swisstph.ch; 7 Basel University, 4001 Basel, Switzerland

**Keywords:** drug-screening, helminth, hookworm, blood-feeding, viability, fluorescence, motility

## Abstract

Hookworm infections cause a neglected tropical disease (NTD) affecting ~740 million people worldwide, principally those living in disadvantaged communities. Infections can cause high morbidity due to their impact on nutrient uptake and their need to feed on host blood, resulting in a loss of iron and protein, which can lead to severe anaemia and impaired cognitive development in children. Currently, only one drug, albendazole is efficient to treat hookworm infection and the scientific community fears the rise of resistant strains. As part of on-going efforts to control hookworm infections and its associated morbidities, new drugs are urgently needed. We focused on targeting the blood-feeding pathway, which is essential to the parasite survival and reproduction, using the laboratory hookworm model *Nippostrongylus brasiliensis* (a nematode of rodents with a similar life cycle to hookworms). We established an in vitro-drug screening assay based on a fluorescent-based measurement of parasite viability during blood-feeding to identify novel therapeutic targets. A first screen of a library of 2654 natural compounds identified four that caused decreased worm viability in a blood-feeding-dependent manner. This new screening assay has significant potential to accelerate the discovery of new drugs against hookworms.

## 1. Introduction

Today, hookworm infections are considered one of the most important causes of a neglected tropical disease (NTD), affecting the lives of ~740 million people [1]. Hookworms mainly infect people living under impoverished conditions in subtropical and tropical regions, particularly those without access to adequate sanitation and footwear. Hookworm disease is characterised clinically by anaemia and malnutrition in pregnant women and children, as well as impaired growth and cognitive development in children [2,3]. Whilst a host immune response to hookworm infection is typically induced, with both humoral and cellular immune responses observed, it fails to afford adequate protection and people tend to exhibit heavier worm burdens with increasing age [4]. To date, no vaccine is available, and preventative chemotherapy to alleviate clinical symptoms is the treatment of choice [4,5].

Albendazole is the main anthelmintic used to treat hookworm infections, and is employed in annual mass drug administration (MDA) programs in endemic areas (with an infection status of ≥40%) to control soil-transmitted helminthiases [6,7,8,9]. Despite an estimated efficacy of 79.5% at reducing hookworm infection [9], in practice, the efficacy of albendazole is variable depending on treatment regimen and community, with a study in Ghana reporting cure rates ranging from 0% to 35% after one dose of 400 mg [10]. Despite this low efficacy, albendazole is administered annually in large-scale treatment programs, raising concerns of emerging drug resistance in endemic areas [11,12]. Indeed, since its introduction to agricultural settings in 1960 to treat nematodes of livestock (e.g., *Haemonchus contortus* and other trichostrongylids), genetic resistance to albendazole has been reported [13,14,15].

Due to the limited treatment efficacy of albendazole in human hookworm populations and lack of new, approved compounds, there is a need for the discovery and development of new and effective treatments and control strategies. However, a substantial barrier to the development of such drugs is the difficulty of obtaining large quantities of hookworm species of humans for experimental work, and the lack of a good laboratory model for pre-clinical studies [16]. Recently, we reported that *Nippostrongylus brasiliensis*, a strongylid of rodents, shares features of its life cycle with the human hookworm *Necator americanus* [17]. *N. brasiliensis* is haematophagous [17], and the blood-feeding/detoxification pathway between *N. brasiliensis* and *N. americanus* is reported to be conserved [17]. Notably, we have utilised this rodent model to identify that quinidine can affect the survival of hookworms by specifically targeting the haem-detoxification required after blood-feeding [17], highlighting this pathway as a potential target for the development of anti-hookworm drugs.

To date, most of the drug screening assays used to identify new anthelmintics take a phenotypic screening approach and measure parasite motility as an end-point [18]. There are several caveats associated with this approach: (i) parasite motility does not always correlate with viability, leading to the identification of drugs with potent activity but that fail when tested for in vivo activity and vice-versa; (ii) parasite motility is often assessed by microscopy, which can be time consuming, low-throughput, and subjective; (iii) the target of the anthelmintic drug is not known *a priori*.

Here, we report a novel semi-high-throughput assay, taking advantage of the shared blood-feeding/detoxification pathway between *N. brasiliensis* and *N. americanus* to identify new drug(s) with nematocidal activity. The assay is a dye-based screen which directly measures parasite viability as an end-point using an automated plate-reader. It can be readily scaled up, does not require extensive training, and is cost-effective and practical. This assay has been designed to be “semi-targeted” in order to identify compounds that disrupt the blood-feeding pathway and associated detoxification processes in the nematode.

## 2. Results

### 2.1. Sytox Green Is a Suitable Dye for the Screening of Drugs Targeting N. brasiliensis

Sytox Green is a nucleic acid stain. Previously, we reported that it is cell membrane and cuticle impermeant for live larvae of the helminth parasite *N. brasiliensis* [19] while able to stain dead larvae, as visualised by microscopy. Here, we assessed whether Sytox Green staining of *N. brasiliensis* could be used in a medium to high-throughput screen as a read-out for viability of the larvae.

**Figure 1:**
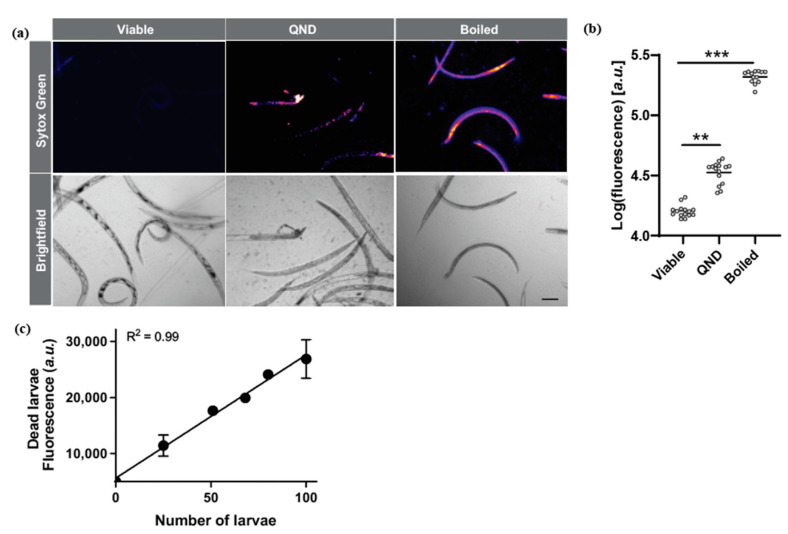
(**a**) Live, quinidine-treated (100 µM, 4 days), or boiled (dead) larvae were exposed to Sytox Green for 24 h prior to microscopic analysis using brightfield or fluorescence emissions. The results were derived from three independent experiments using 100 *N. brasiliensis* iL3s per well. Fluorescence images were analysed using a look-up-table (LUT) “fire” in Fiji (purple low intensity, yellow high intensity). (**b**) Spectrophotometric measurement of Sytox Green. The fluorescence intensity was log10-transformed. Data were pooled from three independent experiments and analysed using the Kruskal-Wallis test. (**c**) Linear regression analysis of the number of dead iL3 and Sytox Green fluorescence, measured 24 h after labelling. Data were pooled from three independent experiments and expressed as mean ± standard error of the mean (SEM) ANOVA were performed and post-hoc significance is indicated, ** *p* < 0.01, *** *p* < 0.001.

We used an in vitro approach in which: (i) live untreated iL3, (ii) iL3 treated with quinidine, or (iii) boiled (dead) iL3 were incubated with Sytox Green for 24 h, after which we assessed the staining of the larvae using microscopic and spectrophotometric approaches (Figure 1a,b, respectively). For the untreated, live iL3s, no specific fluorescence was detected (Figure 1a) and the dye was confirmed to be both cell- and cuticle-impermeant. Fluorescence microscopy of boiled or quinidine-treated iL3s showed that the dye readily passed through the cuticle and stained the larvae with reduced viability (Figure 1a). Spectrophotometric measurement confirmed that the difference in staining between live and dead larvae could be quantitated, with boiled iL3 giving the higher fluorescence (Figure 1b). By serial dilution of boiled iL3s, we showed a strong correlation between fluorescence measurements and the number of dead iL3s (R2 = 0.99, Figure 1c). Variation in results (standard deviations) between, but not within, experiments was high due to inconsistency of the initial viability of the *N. brasiliensis* iL3s. Variation among distinct batches of larvae is expected for such nematodes [20,21].

**Figure 2:**
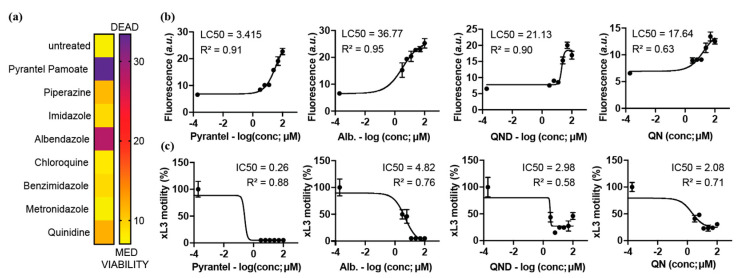
(**a**) *N. brasiliensis* iL3s (*n* = 100) were co-cultured in the presence of RBCs with 100 µM known anthelmintics (pyrantel, piperazine, imidazole, albendazole, chloroquine, benzimidazole, metronidazole and quinidine). After 3 days, Sytox Green was added to the wells and 24 h later, fluorescence (arbitrary units, *a.u.*) was measured by spectrophotometry. The average of duplicate values for each drug are shown as a heat-map ranging from medium viability (MED) to low viability (DEAD i.e., equivalent to killed by boiling) (arbitrary units *a.u.*). (**b**) Drug dilution series for pyrantel, albendazole (Alb.), quinidine (QND), and quinine (QN) using the same assay as described in (**a**). Data were normalised to those for “dead” controls and pooled from three independent experiments and represented as mean ± standard error of the mean (SEM). The compound concentrations were log10-transformed and fitted using a variable slope four-parameter equation, using ordinary least squares fit model. (**c**) Using the same conditions as described in (**b**), motility was assessed by microscopy 4 days after culture. Motility scores were calculated for individual compounds, normalised with reference to the negative control (100% motility) and recorded as percentages. Data points represent one experiment conducted in triplicate; mean ± standard error of the mean (SEM).

We further assessed whether Sytox Green is sufficiently sensitive to use as a viability “readout” for screening compounds against hookworms, by determining the viability of *N. brasiliensis* iL3s using Sytox Green after exposure to eight known anti-parasitic drugs (pyrantel, piperazine, imidazole, albendazole, chloroquine, benzimidazole, metronidazole, or quinidine). As our aim was to identify new compounds targeting the blood feeding pathway of hookworms, *N. brasiliensis* iL3s were incubated in the presence of lysed RBCs to stimulate the feeding and development of worms. Albendazole and pyrantel pamoate are the reference drugs used to cure hookworms in humans or *N. brasiliensis* in rodents. Chloroquine and quinidine have previously been shown to cause a decrease in viability in *N. brasiliensis* in a blood-dependent manner [17]. As previously described, iL3s develop an intestinal pigmentation in response to their hematophagy and enter the third moult after 2 days of in vitro culture (now denoted as ‘L3′) [17]. The compounds of interest were each added at 100 µM to the iL3s. This concentration was established previously for the reference compound, quinidine, which results in the death of 100% *N. brasiliensis* L3 after 4 days of culture [17]. Sytox Green was added after 3 days of drug treatment, and fluorescence was measured 24 h later. In accord with information from published literature [17,20,21,22,23,24,25], all compounds, other than metronidazole, caused an increase in fluorescence as compared with untreated controls (DMSO-only) L3s (Figure 2a). The two most effective compounds identified were albendazole and pyrantel pamoate, anthelmintics both known to be effective against adult hookworms of Ne. americanus and Ni. brasiliensis in vivo and in vitro [22,23,24,26,27,28,29]. As previously described, quinidine was found to be more efficient than chloroquine at decreasing worm viability [17].

To assess the sensitivity of our assay, we further evaluated the lethal concentration 50 (LC50) values for pyrantel, albendazole, quinine, and quinidine (QND) employing the Sytox Green-based assay for viability, and compared the resultant data to the IC50 calculated using the current ‘standard’ assay [30,31] of assessing worm motility (Figure 2b,c). Interestingly but unsurprisingly, the LC50 values were significantly higher than the IC50 values based on motility. Indeed, change in worm motility is usually observed long before killing actually occurs (and thus can be measured by sytox-Green staining), especially in the case of compounds causing paralysis.

### 2.2. A Primary Screen Using Sytox Green Identifies 66 Compounds with Potential Anthelmintic Activity in the Presence of Blood

**Figure 3:**
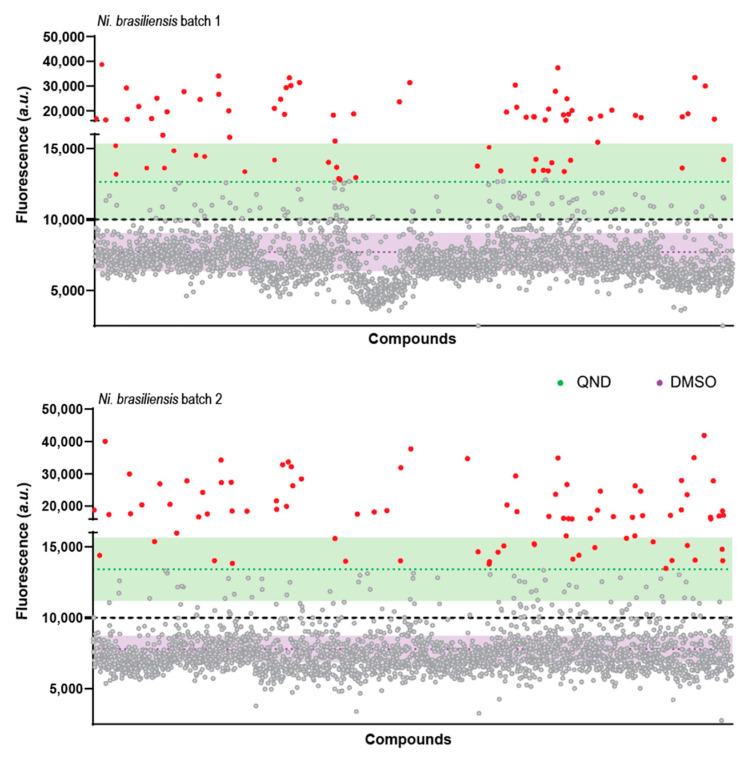
Results of the primary screen of a natural compound library (*n* = 2654) against third-stage larvae of *Ni. brasiliensis* with reference to quinidine as positive control (QND; 32 replicates per plate) and to DMSO 1% as negative control (DMSO, 32 replicates per plate). All compounds were screened in duplicate using different batches of the parasite (top or bottom panel). All values were normalised for each plate to mean (DMSO) + 3 ∗ SD(DMSO) = 10,000 units (Dashed black line). All compounds and positive controls were tested at 100 µM. Each grey or red dot represents an individual test compound. Mean +/− standard deviation (dashed coloured line +/− coloured zone) for controls were calculated as an average of all replicates (*n* = 224) and are indicated in green for QND and purple for DMSO. Red dots are hits identified (as described in Figure S1).

With the goal of identifying new compounds with activity directed against the blood feeding pathway of hookworms, we used a high-throughput assay employing Sytox Green fluorescence detection in a 384-well format with 40 iL3s per well. We screened a library of natural compounds with a broad chemical diversity (put together by the EPFL Bioscreening facility, with compounds from Interbioscreen and Analyticon). The screening of 2650 natural compounds detected 169 and 194 compounds from batch 1 and 2, respectively, which decreased viability of *N. brasiliensis* L3s (Figure 3). Due to the large number of hits using the standard cut-off (mean(DMSO) + 3 ∗ SD(DMSO)), we applied a second cut-off that corresponded to compounds with an activity equal to, or better than, the reference drug—quinidine (QND). All compounds were screened twice using different batches of *N. brasiliensis* to account for the baseline variation in viability reported previously, and only compounds that gave coherent results between repeats were considered further (Figure S1). Altogether, we identified 66 compounds (Figure 3 and Table S1) with potential anthelmintic activity in the presence of blood. For each of the 66 hits detected by Sytox Green-staining, we immediately examined the L3s microscopically for phenotypic alterations in motility and pigmentation in the nematode (Figure S1 and Figure 3).

**Figure 4:**
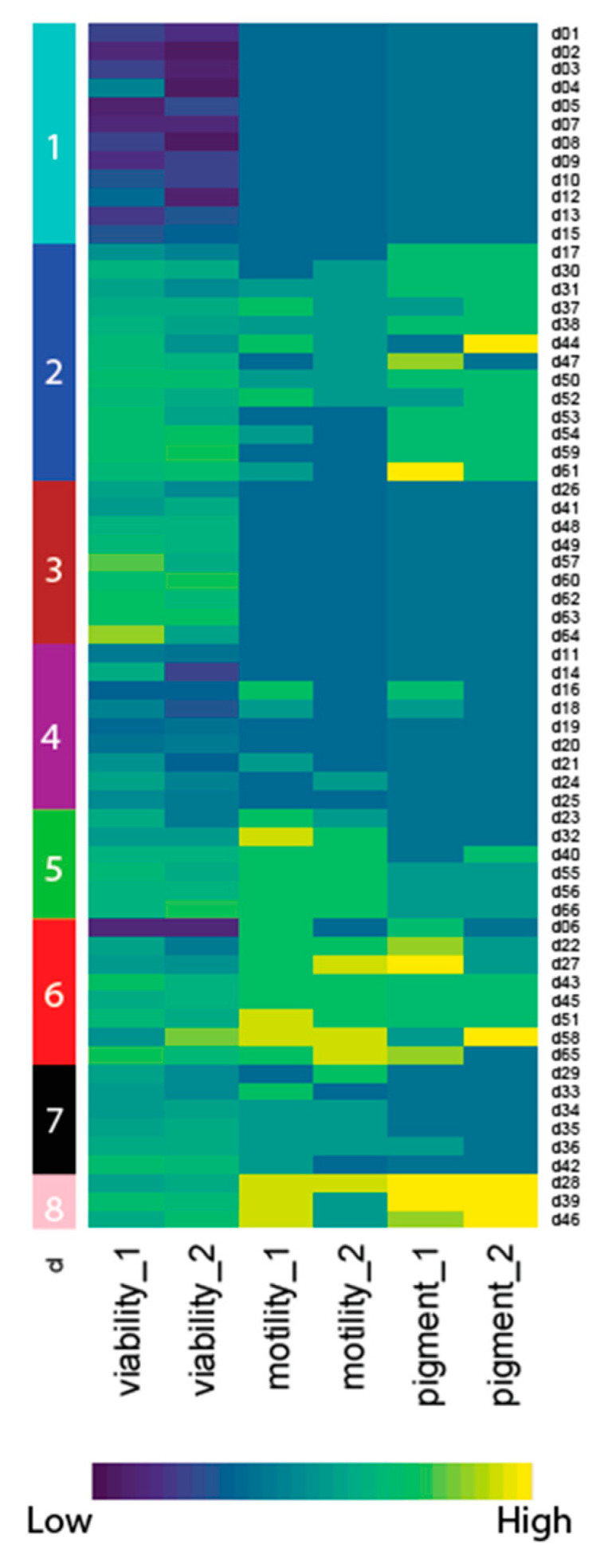
Comparative heat-map of hits displaying scaled readouts for viability, motility, and pigmentation is shown (two duplicates for each drug and two different batches of larvae, “1” and “2” were used). Viability: 40 iL3 per well were incubated with compounds of interest at 100 µM for 4 days. After 72 h, Sytox Green was added to the culture and viability was acquired by spectrophotometric measurement 24 h later. Motility: compounds with an effect that was greater than that obtained for the reference compound (QND) were further scored for motility under bright-field microscopy. Pigmentation: Compounds with an effect that was greater than that obtained for the reference compound (QND) were also scored under bright-field microscopy for the presence/degradation of a hemozoin-like compound. All measurements were performed in the same experiment. Values were Z-scored for each readout (centred of the mean of the data), negative Z-scores (lower fitness) are shown in dark blue, and positive Z-scores (higher fitness) are shown in yellow. Clusters are shown on the left and numbered 1–8.

Interestingly, these three measurements (viability, and follow-up motility and pigmentation assessment) did not always correlate despite being performed on the same parasites (Figure 4, Pearson coefficient of correlation viability/motility 0.360, viability to pigment 0.394, and motility to pigment 0.499). We thus used hierarchical clustering to generate phenotypic clusters, and 4 of these contained a total of 32 compounds that simultaneously reduced all three measurements (i.e., clusters 1, 4, 6, and to a much smaller extent cluster 8), with clusters 1 and 4 containing the most effective compounds (Figure 4, Table S1). Cluster 2 identified 13 compounds that had a stronger effect on motility than on viability and pigmentation, suggesting that they might not be specifically targeting the blood feeding pathway but, instead, cause paralysis. Cluster 7 identified 6 compounds with low pigmentation but with both the viability and motility less affected. This might suggest an interference with the ingestion or digestion of blood, rather than with the associated detoxification pathway.

For compounds in clusters 3 and 5, no correlation between fluorescence-based viability, motility, and pigmentation could be observed. In particular, clusters 3 was enriched for compounds that markedly affected the motility, but not necessarily the viability or the pigmentation. Two compounds from cluster 3 have known anthelmintic activity (#57, an avermectin and #41 calactin, a retonoid). We further confirmed that two of compounds in cluster 3 have in vitro activity against adults of *N. brasiliensis* and against L3 of *N. americanus*, without a strong dependence on blood feeding for activity (data not shown).

### 2.3. Secondary Screen Using Sytox Green Identify 5 Compounds with Specific Blood-Feeding Activity

**Figure 5:**
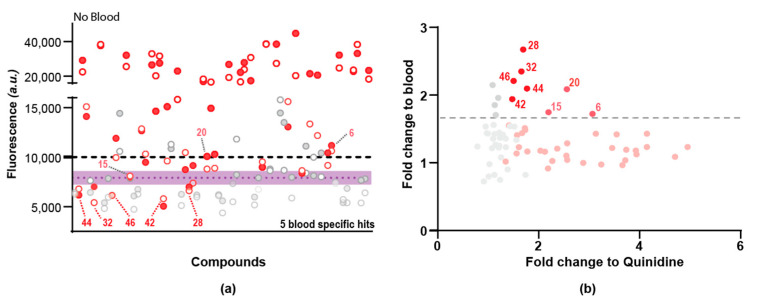
Results of the secondary screen of a natural compound library (*n* = 66) against third-stage larvae *N. brasiliensis* with reference to quinidine as positive control (QND, 32 replicates per plate) and to DMSO 1% as negative control (DMSO, 32 replicates per plate). All test and positive control compounds were tested at 100 µM in the presence or absence of blood in duplicates. Each grey or red dot represents an individual test compound. Mean +/− SD for controls were calculated as an average of all replicates (*n* = 224) and are indicated in purple for DMSO. All values were normalised for each plate to mean (DMSO) + 3 ∗ SD(DMSO) = 10,000 units (Dashed black line). Red dots are compounds identified as hits in presence of blood (39 compounds, screen 2, as described in Figure S1). (**a**) Normalised fluorescence intensity obtained for each of the 66 hits in absence of blood. (**b**) For each of the 66 compounds, the fold change of Sytox green intensity between screen 2 (in presence of blood) and screen 3 (in absence of blood) is represented as a function of the Sytox intensity as compared to the reference drug quinidine in presence of blood. The dash grey line has been set manually to separate hits with the higher fold change between absence and presence of blood.

To determine whether the 66 hits affected L3 viability by interfering with blood-feeding, or whether they targeted an unrelated pathway, we performed a secondary screen on the 66 primary hits, comparing their activity in the presence or absence of RBCs in the culture medium (Figure S1). From these 66 compounds, 39 were confirmed to have anthelmintic activity in the presence of RBCs (Table S2, Figure S2a). Of those 39, 5 compounds had an activity as low or lower than the DMSO control when used in the absence of blood (Table S1, Figure 5a,b). Those 5 blood-specific compounds (namely RA-XII [#42], Anguidine [#44], Emetine [#46], Lycorine [#32], Escin Iva [#28]) all displayed a moderate improvement of efficacy as compared to our blood-specific reference control quinidine. There was no particular enrichment of clusters associated with these specific compounds (Figure S2b). Of interest, three compounds (Bafilomycin A1 [#6], NP-008037 [#15] and Destruxin A [#20]) had relatively low activity in absence of blood (close to the DMSO average) and a strong activity in presence of blood (Figure 5). In total, 17 compounds had an increase in fluorescence score of at least 1.3x between presence and absence of blood (Table S2).

The hits with the highest score rate as compared to quinidine were over-represented in cluster 1 and mostly did not have an activity enhanced in presence of blood (Figure S2b). Phenotypic clusters 1 and 4 both contain known anthelmintics (avermectins, enniatins, and tephrosin), consistent with activities which are not dependent on blood-feeding.

Overall, we observed a general trade-off between compound score rating (as compared to quinidine) and blood specificity, such that compounds with greatest efficacy against the iL3s often exhibited less specificity for the blood-feeding pathway and vice versa (Figure 5b, Table S2).

## 3. Discussion

The biological and molecular complexity of helminth parasites has impaired advances in the design of chemotherapies, with many of the currently available drugs all targeting the parasites’ neuro-muscular pathway. Hookworms are amongst the most common helminth parasites of humans, and cause a characteristic anaemia, due to chronic blood-feeding of worms over a period of years. As blood-feeding is essential to hookworm development, targeting this pathway could both reduce morbidity and lead to parasite elimination from the gut. Used in combination with drugs targeting the neuro-muscular pathway, the risk of emergent drug resistance might be alleviated. With this goal in mind, we report here a newly-developed assay for the identification of anthelmintic compounds, which exhibits two specific features that distinguish it from other reported assays [18,29,30,31,32,33,34]. Firstly, it is able to specifically distinguish compounds that target parasite pathways associated with blood-feeding. Secondly, the assay utilizes fluorescent measurements of parasite viability via cell/cuticle permeability that are readily scalable, compared with traditional, image-based measurements of worm motility, as an assessment of nematocidal activity. Compared to other methods [35,36], the screen we detail here also has no requirement for pre-staining or counterstaining larvae making it simple and cheap to implement, and it can be scaled down to 40 L3 per well, allowing for large number of compounds to be screened with the same parasite batch.

To date, the discovery of new anthelmintics for hookworms using high-throughput assays has been hampered by a lack of ready access to human hookworm species [18]. Thus, screening has been done using related strongylid nematodes [37] or the free-living nematode *Caenorhabditis elegans*, because of the ease with which it can be procured [32,34,38]. However, we should consider that, unlike *C. elegans*, parasitic nematodes such as hookworms have evolved specific pathways of biological importance that allow them to infect and/or thrive in their mammalian host. Indeed, hookworms rely on the blood-feeding pathway for their development, survival, and sexual reproduction [4], rendering it a useful target for chemotherapy or vaccination [39,40]. Here, using a small subset of known anthelmintics, we provide evidence that *N. brasiliensis* larvae can be used for screening libraries of compounds to identify drugs of relevance to human parasitic helminths. Using this methodology, we identified 17 compounds exhibiting enhanced anthelminthic activity in worms actively feeding on RBCs, five of which only exhibit anthelminthic activity for worms actively blood-feeding. As such, the method described here is a clinically relevant way to screen compound libraries.

The use of a viability dye to assess nematode fitness represents a quantitative, objective, and fast read-out that does not require extensive training. As such, it may greatly improve the discovery of novel anthelmintic compounds and can be readily employed to screen compounds for anthelmintic activity unrelated to, or not dependent on, blood-feeding. The present results show that a non-cell permeable dye, such as Sytox Green, allows for quantitative differentiation between viable larvae and larvae with impaired fitness, probably due to cuticle permeability change with events leading to the parasite death. Of note, Sytox green based viability screening could be adapted to nematodes other than hookworms as well as potentially other helminths such as trematodes, if a bright enough dye can be found to overcome the natural autofluorescence of those parasites. Accordingly, this methodology can be scaled up to perform medium- to high-throughput screening assays. A limitation to consider though is the size of the organism, as a requirement for the spectrophotometric measurement to work is that the fluorescence needs to be perceived as “homogenous” rather than “discrete”. As an example, while Sytox Green stain allows for the discrimination of live versus dead adults of Nb, fluorescence quantification by spectrophotometry is not possible as the signal is too localised (microscopy based approached might be though).

Many other viability dyes are available on the market, and some have already been reported to exhibit utility for helminth viability assays [18,33]. Sytox Green has recently been reported as a viability dye for nematodes by us as well as others [19,35,36] and was chosen for the current study based on the absence of toxicity to *N. brasiliensis*, and on its spectrum and brightness, which allows it to be used in an assay in which the medium contains large quantities of the naturally autofluorescent protein, haemoglobin. Haemoglobin is present in two forms: oxygenated and deoxygenated, each with a different absorption spectrum, and we found that the presence of lysed RBCs interfered with the excitation and emission wavelengths of other viability dyes reported in the literature (such as propidium iodide, data not shown) [41].

The present fluorescence-based assay differs substantially from the ‘standard’ for the field of anthelminthic drug development [30,31]. Traditional assays rely on the measurement of parasite motility as a read-out of drug activity on the nematode, based on the assumption that long-term inhibition of parasite motility would result in the eventual starvation of the parasite, or lead to a greater degree of immune-mediated attack and expulsion in vivo. Limitations of this approach include the need for image quantification and/or subjective scoring, and that reduced parasite motility does not necessarily predict larval viability. In our validation experiments (Figure 2), we compared the use of parasite viability versus motility as a readout of drug efficacy using two known anthelmintics, previously shown to be able to kill *N. brasiliensis* in vitro, namely levamisole and pyrantel. Both are cholinergic anthelmintics that act on nematode nicotinic acetylcholine receptors located on somatic muscle cells, which likely prevent muscle contractions, resulting in paralysis and the eventual death of the parasite [42,43,44]. In line with the known ability of these drugs to immobilise nematodes, we observed high LC50 values obtained for the Sytox measurement as compared to IC50 obtained with the motility assay, indicating that a low compound concentration is sufficient to impair the locomotion of the parasite, as previously described for ivermectin in *C. elegans* [41]. Quinidine (QND) and quinine (QN) both interfere with hemozoin formation and kill the parasite in vitro [17]. Once again, concentration to reach LC50 observed for these two compounds using the fluorescence-based viability assay (LC50) were much higher than those observed using a motility-based readout (IC50). This demonstrates a likely difference in sensitivity between the two assays. Importantly, however, both assays revealed that QND exhibited a greater effect than QN.

We believe that the use of a viability assay is an advantage when screening for novel anthelmintics. Nevertheless, a possible disadvantage of employing a fluorescence-based assay to measure cuticle permeability is that some compounds may interfere with the fluorescence of the assay by binding to the dye used, or some compounds emitting fluorescence of the same wavelength as the dye may lead to discrepancies in the estimation of the viability. Therefore, it is necessary that impaired viability is confirmed after screening, which can be achieved using alternative methods such as motility assessment or a qualitative measurement of the hemozoin-like pigment associated with feeding. Due to the time-consuming nature of these follow-up phenotypic assessments, in the present study we chose only to assess those for compounds that were determined to be a hit at the time of making the fluorescence measurement. Indeed, for those compounds for which we observed a marked change in viability (clusters 1, 4, 6, and 8), we also observed a strong correlation among all three parameters (Pearson pairwise comparison, viability, motility, and reduced pigmentation). Phenotypic clusters 1 and 4 both contain known anthelmintics (avermectins, enniatins, and tephrosin), consistent with activities which are not dependent on blood-feeding. An in-depth search of the literature revealed that compounds #41, #46, #47, #51, #56, #57, #58, #61, #65, and #66 have known or likely anthelmintic properties, whilst for the 56 remaining compounds no prior anthelmintic activity has been reported (Table S1). Examples of those with known anthelmintic activity include compound #46, also known as emetine, which was previously employed as an anthelmintic; however, its use was abandoned due to toxicity in the host animal [45]. Similarly, compound #58 is a sesquiterpene lactone related to the anti-malarial drug artemisinin, also known to exhibit anthelmintic activity [46]. These examples further validate the use of Sytox Green as a viability-based assay for the screening of compounds for anthelmintic activity.

Of the 5 compounds identified with activity specific to blood-feeding, 2 were not further considered for evaluation. Anguidine is known to cause toxicity to dividing cells, and as such will not be explored further [47]. Emetine, as its name suggests is an emetic and has reported activity against helminth and amoeba [48]. It has long been used for treatment of amebiasis and fascioliasis, but severe toxicity and adverse events have been reported in humans and the drug use has mostly been discontinued. We are currently validating the other 3 identified compounds further (namely RA-XII, Lycorine, and Escin Iva), as well as a few compounds with activity enhanced by blood-feeding in other stages of *N. brasiliensis* as well as other nematode species.

## 4. Materials and Methods

### 4.1. Preparation of Compounds for Screening

To establish the feasibility of our approach, we first screened a small subset of known anthelmintics and anti-parasitic drugs (namely, pyrantel, piperazine, imidazole, albendazole, chloroquine, benzimidazole, metronidazole, and quinidine) obtained from Sigma Aldrich. We further screened a library of 2650 compounds purified from natural sources originated from plants and micro-organism (bacteria, fungis) and selected for their diversity of structure by the Biomolecular screening facility at EPFL, Lausanne. The compounds have been selected from commercial libraries of Analyticon Discovery GmbH (Potsdam, Germany) (https://ac-discovery.com/natural-resources-and-technologies, accessed on 15 August 2013) and InterBioScreen Ltd. (Moscow, Russia) (https://www.ibscreen.com/natural-compounds, accessed on 15 August 2013). All drugs were prepared and plated at a concentration of 10 mM dimethyl sulfoxide (DMSO).

### 4.2. Rats and Mice

Female Lewis rats (used for maintaining the life cycle of *N. brasiliensis*) and C57BL/6J mice (6–10 weeks of age, used as a source of blood) were obtained from the Charles River facility (Freiburg, Germany) or Jackson laboratory (Ellsworth, Maine, USA) and maintained at École Polytechnique Fédérale de Lausanne (EPFL, Lausanne, Switzerland), at Monash University (Clayton, Victoria, Australia), or at Swiss TPH, Allschwill. All experiments were approved by ethics committees at respective institutions (codes VD-3001, E/1893/2019/M, and BL-526, respectively).

### 4.3. Preparation and Isolation of N. brasiliensis

The life cycle of *N. brasiliensis* (originally provided by Graham LeGros, MIMR, New-Zealand) was maintained as described previously in EPFL or Monash University [19]). Infective third-stage larvae (iL3s) were prepared from faecal cultures performed as described previously [49]. The iL3s were then washed three times in phosphate-buffered saline (PBS) and incubated for 1 h at 37 °C in an antibiotic solution (penicillin/streptomycin 1000 U/mL (Gibco), gentamicin 300 U/mL (Sigma) in PBS) prior to use in screening assays.

### 4.4. Conditions of Larval Culture with All Compounds

Larvae were cultured in complete DMEM medium (Gibco) supplemented with 10% FBS (Gibco), 2 mM L-glutamine (Gibco), 100 U/mL penicillin/streptomycin (Gibco), 0.5 mg/mL gentamicin (Sigma), 10 mg/mL tetracycline (Gibco).

Red blood cells (RBC) were collected by cardiac puncture or cheek vein bleeding into Alsevers solution (2.05% dextrose (Sigma), 0.8% sodium citrate (Sigma), 0.055% citric acid (Sigma), and 0.42% sodium chloride (Sigma) in distilled H_2_O) on the day of culture. RBCs were washed three times in RBC-wash buffer (containing 21.0 mM tris(hydroxymethyl)aminomethane (Sigma), 4.7 mM KCl (Sigma), 2.0 mM CaCl2 (Sigma), 140.5 mM NaCl (Sigma), 1.2 mM MgSO_4_ (Sigma), 5.5 mM glucose (Sigma), and 0.5% bovine albumin fraction V; pH 7.4 (Sigma)) [17], resulting in 98% purity (as identified by Ter119-staining (Life-technologies) by flow cytometry), and lysed in water before being added to 10X concentrated DMEM (Gibco) at 1 × 10^8^ cells/mL.

The iL3s (*n* = 100 or 40) were co-cultured in 100 µL (for pre-screening experiments, Figure 1 and Figure 2) or 40 µL (for primary and secondary screens, Figure 3, Figure 4 and Figure 5) of medium in 96- and 384-well plates, respectively, with test and positive controls (Quinidine) compounds at 100 µM or no compound (i.e., negative (DMSO-only) control), and incubated for 4 days at 37 °C, 5% CO_2_. Dead larvae were prepared by incubating a dry pellet of larvae (about 100 µL in a 1.5 mL Eppendorf) for 5 min at 90 °C.

For the natural compounds screen, all compounds were screened in duplicates on two separate plates, using two batches of *Ni. brasiliensis* to account for the biological variability of the parasite. A total of 32 replicates of 1% DMSO only and 32 replicates of Quinidine 100 µM were included in each plate to assess the robustness of the assay. Of note, 1% DMSO has not been found to impact the viability of the larvae.

### 4.5. Sytox-Green Fluorescence Assessment

In pre-screen experiments, Sytox Green fluorescence together with bright field was assessed on an Olympus System CellR at the Bioimaging and optics platform (BIOP) in EPFL.

In both pre-screen experiments and for the natural compounds screen on the third day of culture, Sytox Green (Invitrogen) was added to a final concentration of 50 µM (1:100) and spectrophotometric measurements were taken 24 h later using an automated microplate fluorometer (Tecan Infinite F500, Tecan Group Ltd. (Männedorf, Switzerland) or FLUOstar Omega, BMG Labtech GmbH, Mornington, Victoria, Australia) using an excitation/emission measurement (504 nm/523 nm).

### 4.6. Pigmentation Assessment

For the natural compounds screen, on the fourth day of culture, the presence/absence of haemozoin-like pigment was evaluated by light microscopy and scored according to intensity; 0 = no pigment, 1 = light brown, non-continuous pigmentation, 2 = light brown or darker pigment, continuous pigmentation, 3 = dark brown pigmentation.

### 4.7. Motility Assessment

In pre-screen experiments, to estimate half-maximal inhibitory concentration (IC50) values, an automated motility assay was used. Briefly, after 4 days of incubating iL3s with individual compounds in a 96-well plate format in triplicate, motility was assessed as described previously [30,31]. Plates were agitated at 126 rotations per minute (rpm) using an orbital shaker for 15 min at 38 °C and 10% *v*/*v* CO_2_. The motility in each well was recorded by video of the well for 5 s using a rate of 10 frames per second employing a grey-scale camera (Q-Imaging Rolera bolt, Surrey, British Columbia, Canada) attached to a stereomicroscope (Olympus SZ61 stereomicroscope, Tokyo, Japan) with Ludl BioPoint 2 motorised-stage (BioImaging Solutions Inc., San-diego, CA, USA). Every 4 min (half a plate), the plate was re-agitated for 15 min at 38 °C. Each video was processed using a customized macro in the program ImageJ (version 2.0.0-rc-69/1.52n, Fiji, https://imagej.net/software/fiji/ accessed on 15 August 2013) to translate changes in the light intensity into a motility-index [30,31].

For the natural compounds screen, on the fourth day of culture, for the screening of natural compounds, motility was assessed by scoring in detected hits (based on their Sytox Green measurement, see Figure S1). Motility was scored using a bright-field microscope with 4X objective as follows; 0 = 90–100% non-motile, partially degraded, 1 = 70–90% of the larvae were non-motile, 2 = 50–70% of the larvae were non-motile, 3 = reduced motility in at least 50 % of larvae, 4 = motile (DMSO-only controls).

### 4.8. Hit Identification

For the primary screen of the natural compound library (screen 1, Figure S1), compounds were defined as hits if the fluorescence obtained was higher than the mean + 3 standard deviations (μ + 3σ) of the fluorescence obtained for DMSO alone. Due to a large number of hits obtained using this threshold, we applied a second cut-off that corresponded to the average Sytox Green fluorescence obtained for the positive control quinidine on each screened plate (Figure S1).

For the secondary screens (screens 2 and 3) with which we wished to identify compounds that acted in a blood-feeding specific manner, we screened the 66 hits that were identified in the primary screen for both replicates (Figure S1). Those 66 hits were then assessed for their impact on worm viability (using fluorescence measurement) in presence (screen 2) and in absence of blood (screen 3). In screen 2, hits were identified as higher than the mean + 3 standard deviations (μ + 3σ) of the fluorescence obtained for DMSO alone. Blood-feeding specific hits were further defined in screen 3 as inferior to the mean of the fluorescence obtained for DMSO alone (Figure S1).

### 4.9. Statistical Assessment of the Assay Robustness

To measure assay quality, 32 replicates of positive (quinidine, QND) and negative (DMSO) control data were used on each 384-well plate screened to calculate Strictly Standardized Mean Deviation (SSMD) for each screening plate using the equation:

$$\beta =\frac{\left|\bar{\mu_{c}}-\bar{\mu_{s}}\right|}{\sqrt{\sigma_{c}^{2}+\sigma_{s}^{2}}}$$

where σ is the standard deviation and μ the mean. Subscript “s” indicates “sample” (i.e., compound to be tested) and “c” stands for “control” (i.e., DMSO alone). We could not use the z’ factor, classically used for drug screening, as Sytox Green readings from healthy DMSO-treated iL3 did not follow a Gaussian distribution. The signal to noise ratio values ranged from 7.66 to 11.38. The plate SSMDs ranged from 4.38 to 6.31. These results demonstrate the robust performance of the assay [50].

### 4.10. Phenotypic Clustering

Data issued from the primary screen of the natural compound library were analysed in R (v4.0.3). Sytox Green fluorescence viability measurement was rescaled so that lower values correspond to lower fitness, as with readouts of motility and pigmentation. Observations from both replicates were collated and processed in a Principal Components Analysis (variables centred and scaled to unit variance, function PCA in package FactoMineR v2.3), and no evidence of a replicate effect was found. We also added the average readouts for each drug to the PCA, to stabilise the following step. We performed hierarchical clustering (function hclust, method = ”ward.D2”) on the observation coordinates (Euclidean distance) for all three principal components, and cut the resulting tree using an automated approach (function cutreeDynamic, package dynamicCutree v1.63-1, minClusterSize=5 and with provided dissimilarity matrix), leading to 8 “phenotypic” clusters. Of 66 drugs, #43 had both replicates and their cross-replicate average clustered in the same cluster. To assign the other drugs to a unique cluster, they were assigned to the cluster to which their mean point belonged.

### 4.11. Other Statistical Analysis

The choice of statistical tests was based on sample size and on Bartlett’s test when normal distributions of the errors were expected. Data from separate experiments were pooled when possible. Statistical parameters including the exact value of n, the definition of centre, dispersion, and precision measures (mean ± SEM) and statistical significance are reported in the figures and/or figure legends.

Representation and data analysis were performed GraphPad Prism 8.2.1. Statistically significant values are indicated as follows: *p* > 0.05; * *p* < 0.05; ** *p* < 0.01; *** *p* < 0.001.

## 5. Conclusions

In conclusion, we report a novel assay for use in the discovery of new chemical entities active against hookworms. The use of a fluorescent dye to measure viability renders the assay practical, cost effective, sensitive, and readily scalable. Applications outside of drug-screening could also be proposed for this method, including gene knockdown or immunological assays to measure the survival of the parasite. Importantly, it should also be possible to adapt the assay for industrial settings, notably with the robotization of parasite distribution into multi-well plates. Furthermore, the ability to screen for compounds that target the blood-feeding pathway of parasitic nematodes offers the potential to identify novel compounds and will likely accelerate the identification of hit, and eventually, lead candidates. Although this assay was developed specifically for hookworms it may also prove useful for the identification of novel anthelmintics active against other blood-feeding nematodes such as *Haemonchus contortus* and *Schistosoma* spp. [39,40]. In summary, the present investigation supports efforts toward finding improved treatments against hookworm infection, focused on reducing infection-related morbidity for up to 0.7 billion people worldwide, and providing significant and immediate economic benefits in terms of human health and well-being.

## Data Availability

Data are contained within the article and Supplementary Material.

## Supplementary Materials

The following are available online at https://www.mdpi.com/article/10.3390/ph15060669/s1, Figure S1: Work-flow of the screening of 2650 natural compounds, Figure S2: Secondary screen scatter plots, Table S1: List of the 66 compounds identified as hits in screen 1, Table S2: List of 39 compounds identified as blood-specific dependent or not from screens 2 and 3.
