# Supplementary material for: Novel High-Throughput Fluorescence-Based Assay for the Identification of Nematocidal Compounds That Target the Blood-Feeding Pathway
Source: Pharmaceuticals (Basel). 2022 May 27;15(6):669. doi: 10.3390/ph15060669 (PMC9231213; doi:10.3390/ph15060669)
Supplement: Supplementary file 1 [file pharmaceuticals-15-00669-s001.zip › pharmaceuticals-1721436 - Supplementary/FigureS1.pdf]

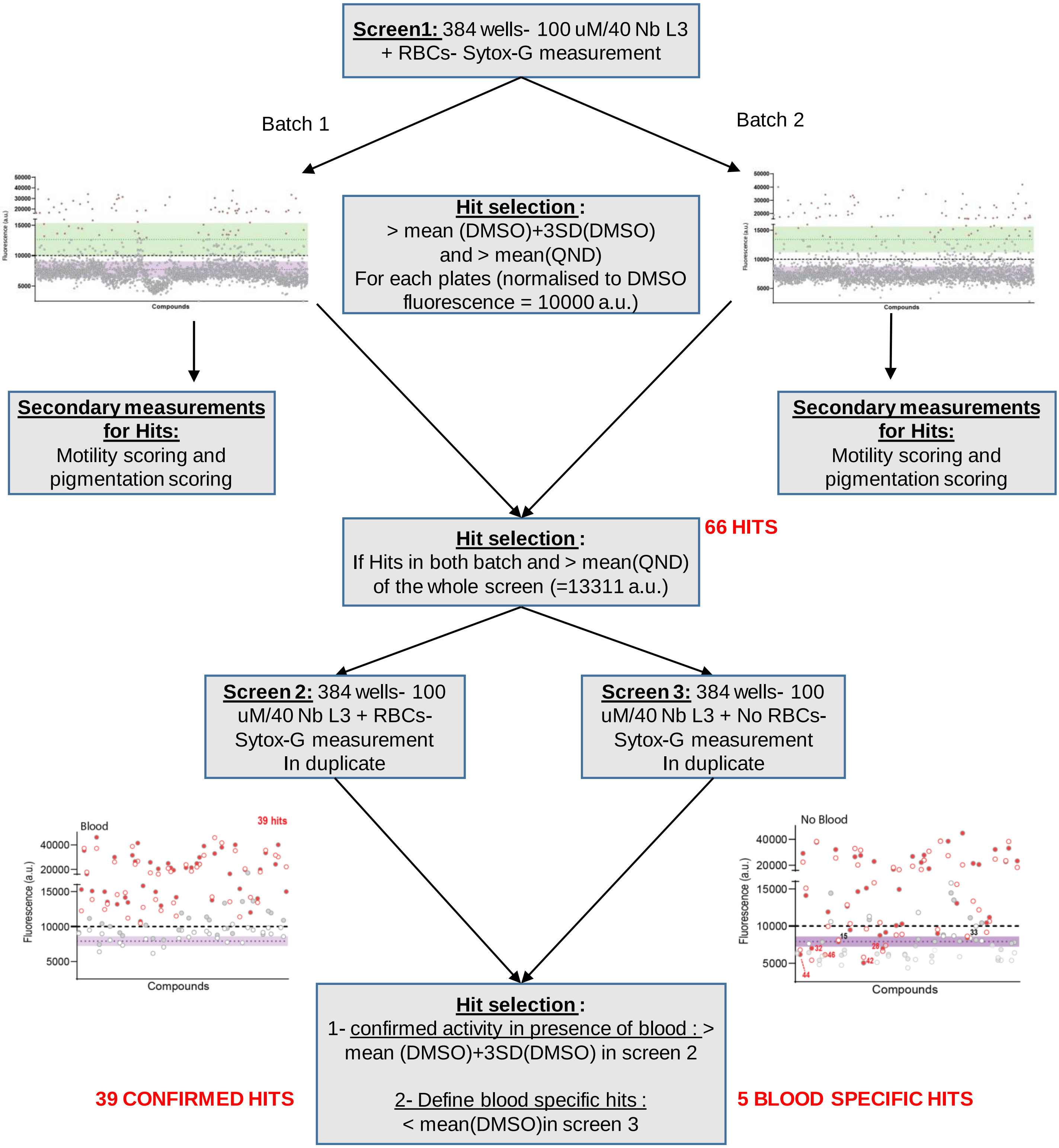

Figure S1. A schematic of the compound screening pipeline is presented. *Ni. brasiliensis* L3 were incubated in presence of 100 uM of compounds and sytox-Green fluorescence was measured 4 days post-incubation. Screen 1 was run in duplicates (batch 1 and 2). Hit detection threshold were applied for each plate and each batch resulting in the identification of 66 hits from Screen 1. On those 66 hits, motility and pigmentation were scored. A second and third screen were performed on those 66 hits in presence or absence of blood. In screen 2, 39 compounds were confirmed as hits. In screen 3, 5 compounds were identified with no activity in absence of blood (while they had an activity in presence of blood).
