# Supplementary material for: Novel High-Throughput Fluorescence-Based Assay for the Identification of Nematocidal Compounds That Target the Blood-Feeding Pathway
Source: Pharmaceuticals (Basel). 2022 May 27;15(6):669. doi: 10.3390/ph15060669 (PMC9231213; doi:10.3390/ph15060669)
Supplement: Supplementary file 1 [file pharmaceuticals-15-00669-s001.zip › pharmaceuticals-1721436 - Supplementary/FigureS2.pdf]

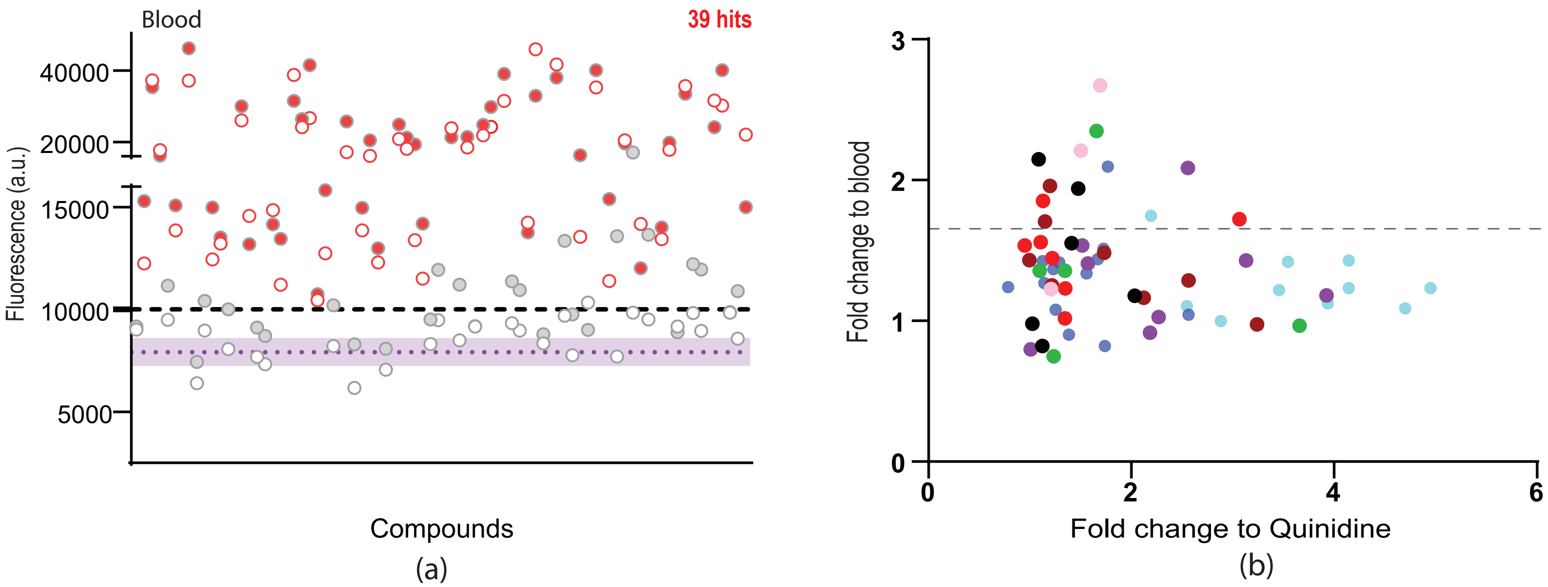

Figure S2. Results of the second and third screen of a natural compound library (n = 66) against third-stage larvae Ni. brasiliensis with reference to quinidine as positive control (QND, 32 replicates per plate) and to DMSO 1% as negative control (DMSO, 32 replicates per plate). All test and positive control compounds were tested at 100µM in presence or absence of blood in duplicates. Each grey or red dot represents an individual test compound. Mean +/- SD for controls were calculated as an average of all replicates (n=224) and are indicated in purple for DMSO. All values were normalised for each plate to mean (DMSO)+3*SD(DMSO) =10 000 units (Dashed black line). Red dots are compounds identified as hits in presence of blood (39 compounds, screen 2, as described in Figure S1). (a) normalised fluorescence intensity obtained for each of the 66 hits in presence of blood. (b) For each of the 66 compounds, the fold change of Sytox green intensity between screen 2 (in presence of blood) and screen 3 (in absence of blood) is represented as a function of the sytox intensity as compared to the reference drug quinidine in presence of blood. Appartenance to a specific compounds is represented based on the colors defined in Figure 4.
