# Supplementary material for: Novel High-Throughput Fluorescence-Based Assay for the Identification of Nematocidal Compounds That Target the Blood-Feeding Pathway
Source: Pharmaceuticals (Basel). 2022 May 27;15(6):669. doi: 10.3390/ph15060669 (PMC9231213; doi:10.3390/ph15060669)
Supplement: Supplementary file 1 [file pharmaceuticals-15-00669-s001.zip › pharmaceuticals-1721436 - Supplementary/TableS1.pdf]

Drug name	DrugID	Cluster #
Niphimycin Ia	d01	1
avermectinB1a	d02	1
Emerimicin IV	d03	1
Guanidylfungin A	d04	1
Heptaibin	d05	1
Enniatin B	d07	1
Asteltoxin	d08	1
Enniatin B1	d09	1
Emamectin B1a	d10	1
Rotenolone	d12	1
Tephrosin	d13	1
NP-008037	d15	1
NP-018353	d17	2
NP-018355	d30	2
STOCK1N-67126	d31	2
STOCK1N-52522	d37	2
Deoxyschizandrin	d38	2
Anguidine	d44	2
NP-011181	d47	2
NP-017509	d50	2
STOCK1N-66495	d52	2
NP-008035	d53	2
NP-018356	d54	2
NP-017678	d59	2
STOCK1N-53266	d61	2
NP-013681	d26	3
Calactin	d41	3
STOCK1N-56995	d48	3
Tabersonine	d49	3
2,3,5,7,8,15-Hexahydroxy-6(17),11-jatrophadiene-9,14-dione, 8-(2-Methylpropanoyl), 2,3,5…	d57	3
STOCK1N-75069	d60	3
11-Hydroxytephrosin	d62	3
STOCK1N-68379	d63	3
Wogonoside	d64	3
NP-018349	d11	4
NP-001014	d14	4
Asclepin	d16	4
STOCK1N-53959	d18	4
Eprinomectin B1a	d19	4
Destruxin A	d20	4
Bafilomycin C1	d21	4
Cyclooctatin	d24	4
Manassantin A	d25	4
8,15-Labdanediol	d23	5

STOCK1N-67168	d32	5
NP-016399	d40	5
NP-006714	d55	5
NP-008036	d56	5
STOCK1N-53835	d66	5
Bafilomycin A1	d06	6
NP-010957	d22	6
Maejaposide	d27	6
NP-017512	d43	6
STOCK1N-52768	d45	6
NP-006392	d51	6
Asteriscunolide A	d58	6
Colchicine	d65	6
Tunicamycin	d29	7
Manassantin B	d33	7
STOCK1N-38480	d34	7
STOCK1N-49640	d35	7
STOCK1N-36398	d36	7
RA-XII	d42	7
Escin IVa	d28	8
NP-018412	d39	8
Emetine	d46	8

Table S1. List of 39 compounds identified as hits from screen 1 and categorized by clustering.
