# Supplementary material for: Novel High-Throughput Fluorescence-Based Assay for the Identification of Nematocidal Compounds That Target the Blood-Feeding Pathway
Source: Pharmaceuticals (Basel). 2022 May 27;15(6):669. doi: 10.3390/ph15060669 (PMC9231213; doi:10.3390/ph15060669)
Supplement: Supplementary file 1 [file pharmaceuticals-15-00669-s001.zip › pharmaceuticals-1721436 - Supplementary/TableS2.pdf]

Drug name	Drug ID	Cluster #	SPECIFICITY<br>Fold change with blood feeding	RATING<br>Fold change to QND
STOCK1N-67126	31	2	0.83	1.74
STOCK1N-53959	18	4	0.92	2.18
8,15-Labdanediol	23	5	0.97	3.66
Wogonoside	64	3	0.98	3.24
Tephrosin	13	1	1.00	2.88
NP-010957	22	6	1.02	1.35
NP-018349	11	4	1.03	2.27
STOCK1N-65883	10	1	1.04	3.71
STOCK1N-52522	37	2	1.04	2.56
Guanidylfungin A	4	1	1.09	4.70
Rotenolone	12	1	1.11	2.55
Emerimicin IV	3	1	1.13	3.94
NP-013681	26	3	1.16	2.12
Tunicamycin X	29	7	1.18	2.03
NP-001014	14	4	1.18	3.92
STOCK1N-67743	2	1	1.19	3.73
STOCK1N-63866	19	4	1.21	2.37
Asteltoxin	8	1	1.22	3.46
STOCK1N-52768	45	6	1.23	1.35
Enniatin B	7	1	1.23	4.95
Niphimycin Ia	1	1	1.23	4.14
ENHANCED BY BLOOD FEEDING				
Calactin	41	3	1.29	2.56
NP-018356	54	2	1.34	1.56
Manassantin A	25	4	1.41	1.57
Enniatin B1	9	1	1.42	3.54
Asclepin	16	4	1.43	3.13
Heptaibin	5	1	1.43	4.14
Tabersonine	49	3	1.48	1.73
NP-018353	17	2	1.51	1.73
Manassantin B	33	4	1.55	1.41
Bafilomycin A1	6	6	1.72	3.07
NP-008037	15	1	1.75	2.19
Destruxin A	20	4	2.09	2.56
BLOOD FEEDING DEPENDENT				
**RA-XII**	**42**	**7**	**1.94**	**1.48**
**Anguidine**	**44**	**5**	**2.10**	**1.77**
**Emetine**	**46**	**8**	**2.21**	**1.50**
**Lycorine**	**32**	**2**	**2.35**	**1.66**
**Escin IVa**	**28**	**8**	**2.67**	**1.69**

Table S1. The requirement for blood feeding as part of a mode of action was determined by comparing the efficacy of the compounds to decrease Sytox fluorescence in the presence or absence of RBCs. The results show fold-change for the compound activity in presence of blood feeding (specificity: L3 viability in the presence of the drug with *versus* without RBCs) with conditional formatting (higher and lower values in red and white respectively) and fold-change for the drug activity as compared to QND (Potency) with conditional formatting (higher and lower values in green and white respectively). Mean of 2 fold-changes of triplicate measurements are shown. Of note, only 38 compounds are shown, as Maejaposide could not have a fold-change calculated as the fluorescence intensity was above the upper limit of detection.
